# Supplementary material for: Evolution of metazoan oxygen-sensing involved a conserved divergence of VHL affinity for HIF1α and HIF2α
Source: Nat Commun. 2019 Jul 23;10:3293. doi: 10.1038/s41467-019-11149-1 (PMC6650433; doi:10.1038/s41467-019-11149-1)
Supplement: Supplementary file 1 — Supplementary Information [file 41467_2019_11149_MOESM1_ESM.pdf]

Evolution of metazoan oxygen-sensing involved a conserved divergence of VHL affinity for HIF1α and HIF2α

Tarade et al.

Supplementary Information

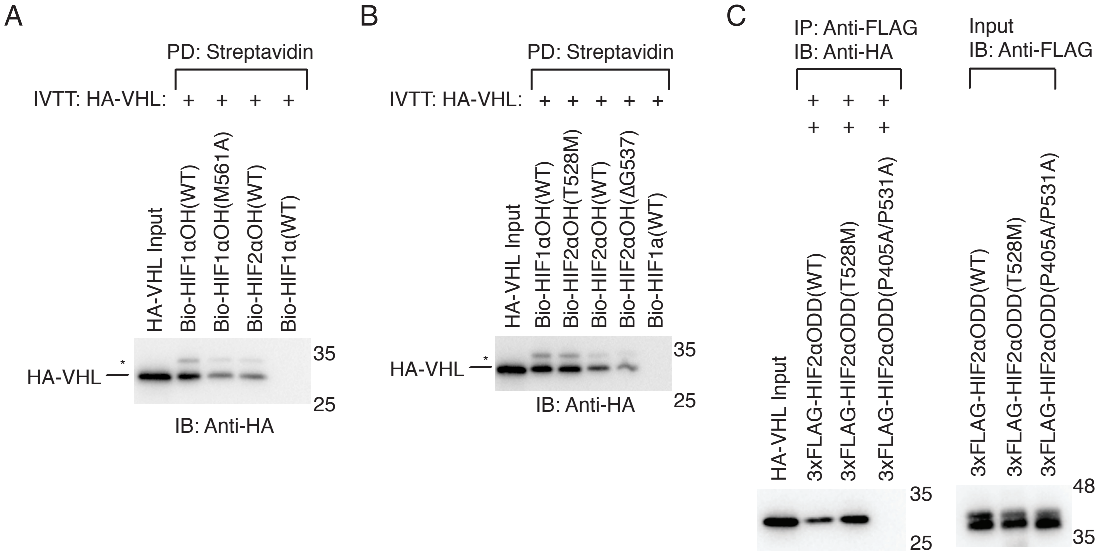

Supplementary Figure 1. Substitution of Met$_{n-3}$ with alanine or threonine results in decreased affinity for pVHL. a, b Biotinylated HIFαOH peptides were immobilized on streptavidin- agarose beads and incubated with *in vitro* transcribed and translated (IVTT) pVHL. Streptavidin beads were pulled down (PD) and levels of HA-tagged pVHL were visualized via immunoblotting (IB). **c** 3xFLAG-HIF2α oxygen dependent degradation (ODD) domains were IVTT and incubated with purified HIS$_6$-PHD2 (181-426). Following hydroxylation (one hour), 3xFLAG-HIF2α ODD domain was immobilized on protein A beads coated with anti-FLAG antibody and incubated with IVTT HA-VHL. 3xFLAG-HIF2α ODD domain was immunoprecipitated (IP) and levels of HA-tagged VHL were visualized via immunoblotting (IB). Molecular weight markers (kDa) are labeled.

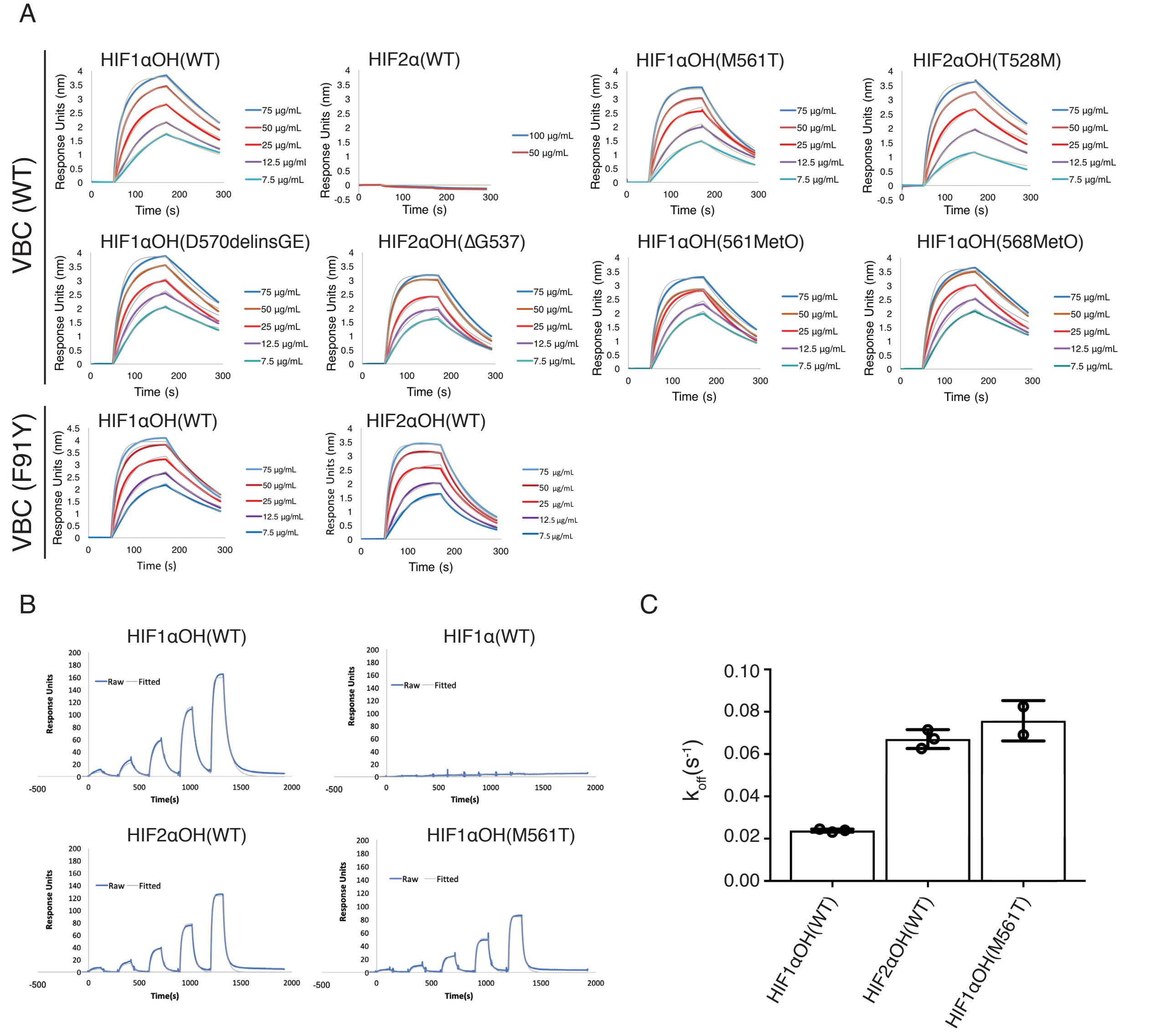

Supplementary Figure 2. Kinetic analysis of VHL complex binding to HIFα peptides**. a** Biolayer interferometry kinetic analysis of pVHL-elongin B-elongin C (VBC), either WT or F91Y (where indicated), complex binding to biotinylated HIFαOH peptides. Biotinylated peptides were coupled to streptavidin- coated biosensors and monitored for binding to VBC complex at the indicated concentrations. The data were analyzed based on a 1:1 binding model using the BLItz Pro software with the fitted curves shown as gray lines. Sensorgrams are representative of three experiments conducted with independently purified proteins. **b, c** Surface plasmon resonance analysis of VBC(WT) complex binding to biotinylated HIFαOH peptides. Biotinylated peptides were coupled to a streptavidin- coated SPR chip. Single-cycle kinetics were carried out with increasing concentrations of VBC (0.123 µg/mL, 0.37 µg/mL, 1.11 µg/mL, 3.33 µg/mL, 10 µg/mL) with a contact time of 120 seconds and a final dissociation step of 600 seconds. A flow rate of 30 µL/min was maintained throughout the experiment. The data were analyzed based on a 1:1 binding model using the Biacore X100 evaluation software with the fitted curves shown as gray lines. **b** Representative sensorgrams. **c** The dissociation constants associated with VBC binding to HIFα peptides are shown on a linear scale. Values represent mean ± s.d. Two technical replicates were performed for each peptide with an additional replication performed with independently purified protein for HIF1αOH(WT) and HIF2αOH(WT).

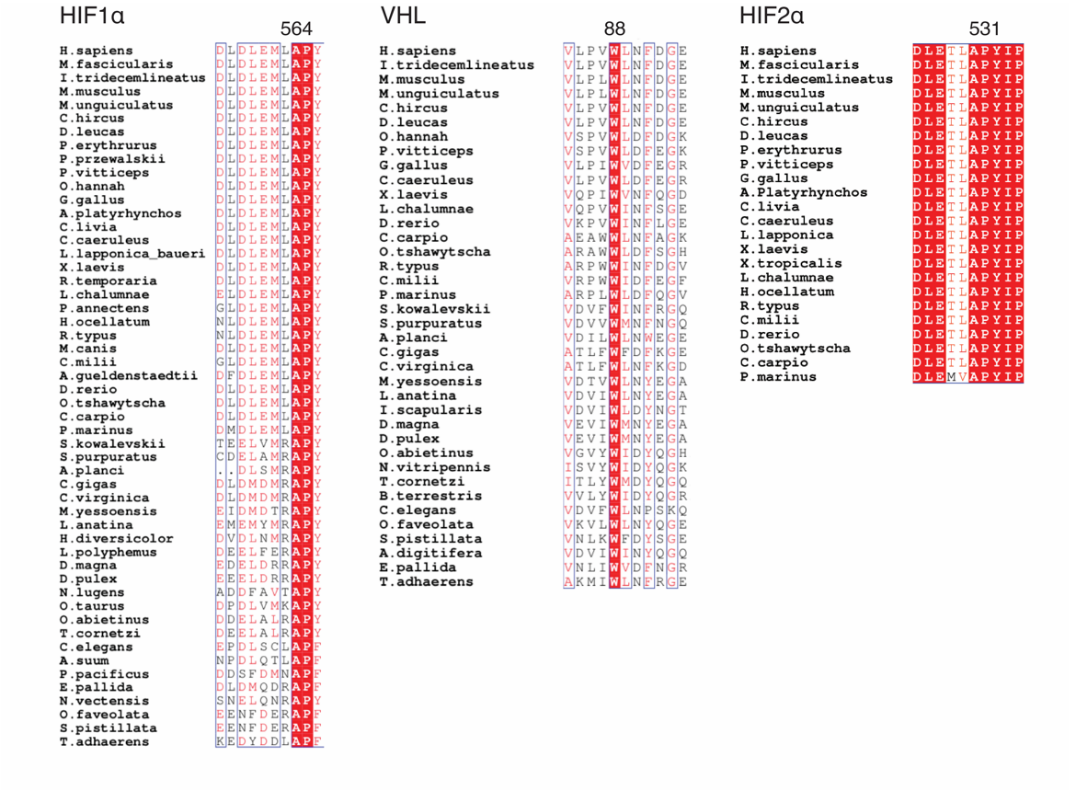

Supplementary Figure 3. Alignment of amino acid sequences. Annotated HIF1α, VHL, and HIF2α sequences were identified through use of BLASTP. Sequences were aligned using the MAFFT algorithm via the GUIDANCE webserver. Numbering of residues is based on the *H. sapiens* protein. Columns where >70% of residues are equivalent are colored in red and boxed in blue. Invariant residues are colored white on a red background. ESPript3 was used to display alignments.

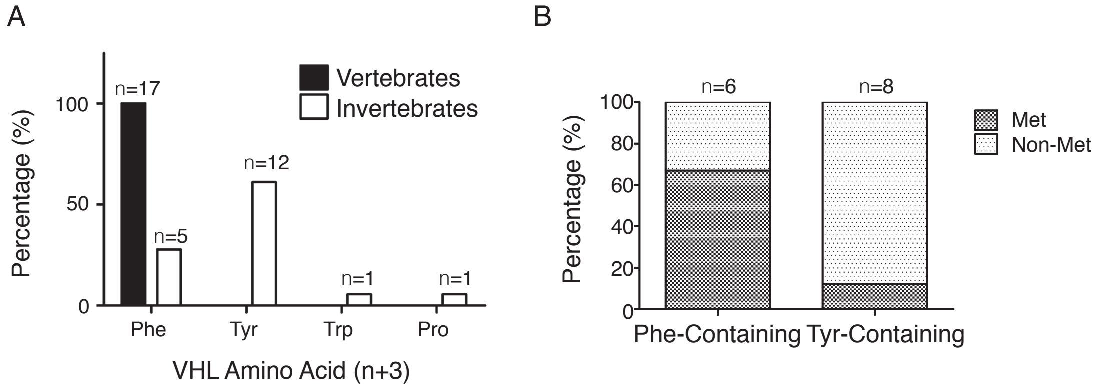

Supplementary Figure 4. VHL Phe_{n+3} is conserved in vertebrate species. a Annotated VHL sequences were identified through use of BLASTP. Sequences were aligned using the MAFFT algorithm via the GUIDANCE webserver. The frequency of the amino acid three residues C-terminal of an invariant tryptophan (VHL Trp88 in humans) is indicated for vertebrate and invertebrate species. **b** Based on VHL and HIFα sequence alignments, the frequency of VHL Phe_{n+3} and HIFα Met_{n-3} are indicated. As VHL Phe_{n+3} and HIF1α Met_{n-3} are invariant in vertebrate species, the analysis is only conducted in invertebrate species for which an annotated VHL and HIFα sequences are available.

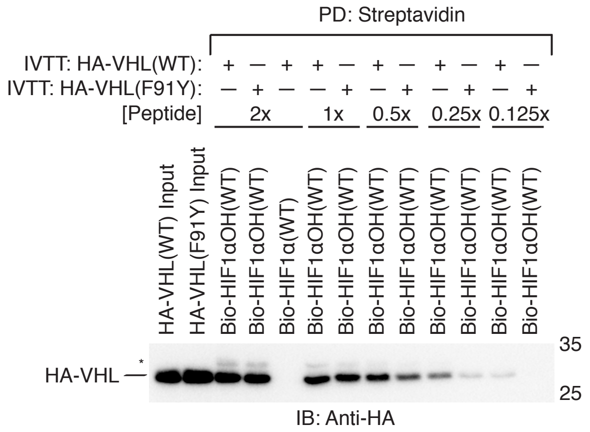

Supplementary Figure 5. Increased peptide concentration obscures differential binding of WT and F91Y VHL to HIF1αOH. 1x peptide concentration = 1.2 µg. Biotinylated HIFαOH peptides were immobilized on streptavidin-agarose beads and incubated with in vitro transcribed and translated (IVTT) pVHL. Streptavidin beads were pulled down (PD) and levels of HA-tagged pVHL were visualized via immunoblotting (IB). Molecular weight markers (kDa) are labeled.

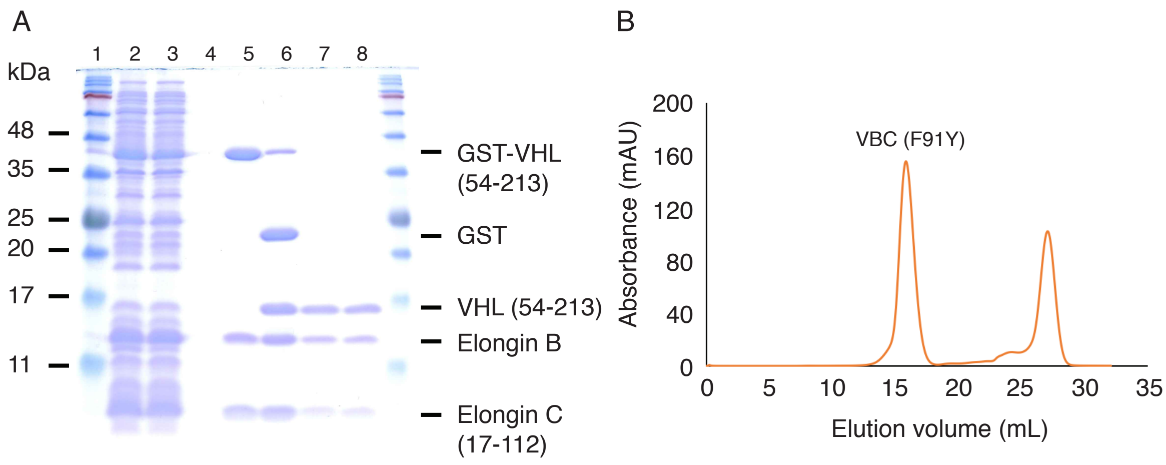

Supplementary Figure 6. Purification of pVHL(F91Y)-elongin B-elongin C (VBC) complex. Transformed BL21(DE3) *E. coli* were induced to express GST-tagged $pVHL_{19}$ (residues 54-213) along with untagged elongin B and elongin C (17-112). (A) GST-VBC complex was affinity purified using glutathione sepharose resin. Thrombin was used to cleave the GST-tag from pVHL. Affinity purification was used to remove GST from the protein solution. SDS-PAGE analysis followed by Coomassie staining was employed to monitor successful cleavage of the GST-tag and purity of the sample. Lane 1 = protein ladder; lane 2 = input; lane 3 = flow-through; lane 4 = wash; lane 5 = elution; lane 6 = cleavage; lane 7 = affinity purification; lane 8 = 2 µg purified VBC following size exclusion chromatography (SEC). (B) VBC (F91Y) complex was purified to homogeneity using SEC. The VBC (F91Y) complex had an elution volume of approximately 16 mL. mAU = milli absorption units.

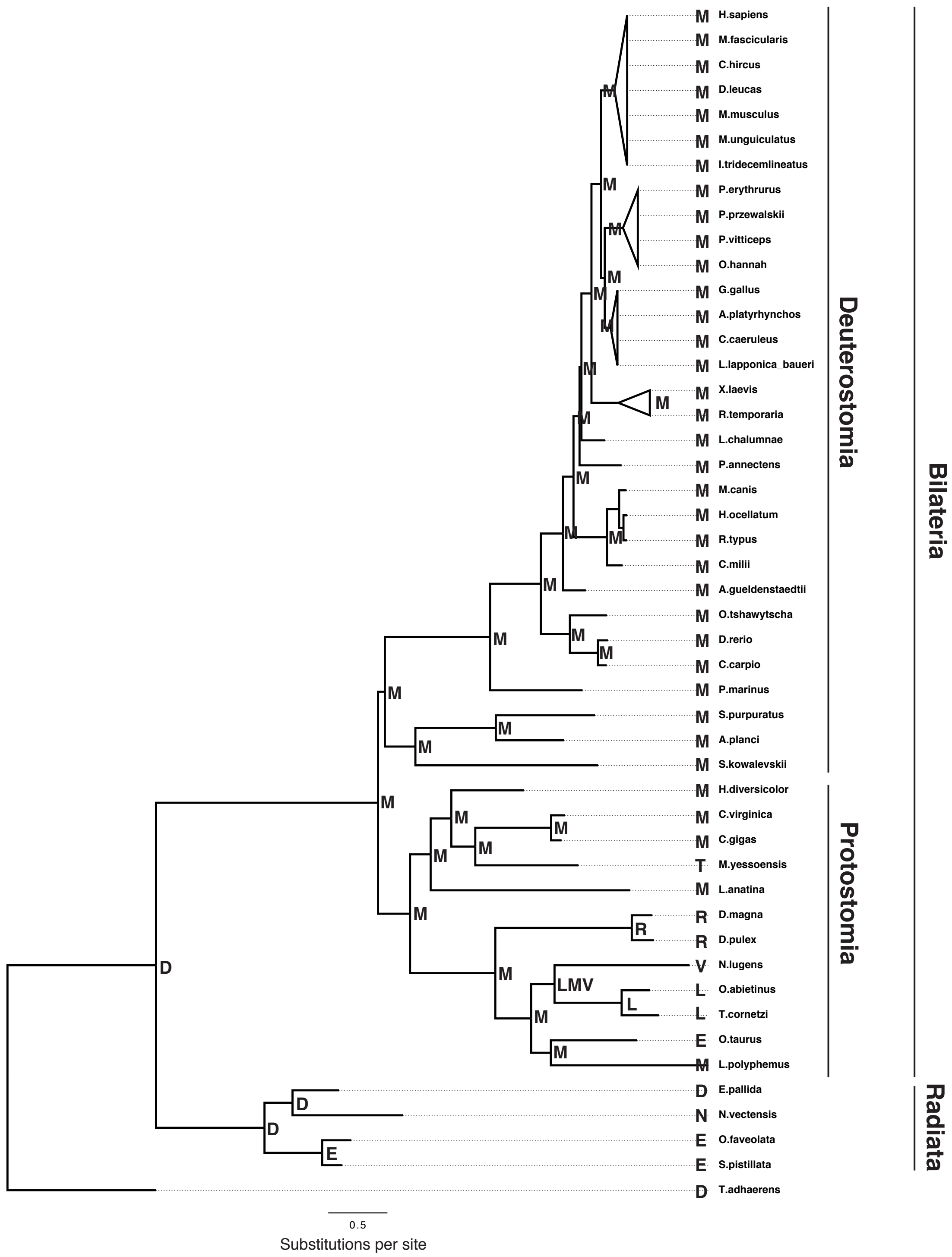

Supplementary Figure 7. Inference of ancestral HIF1α sequence. Using a maximum parsimony method, the amino acid identity of HIF X_{n-3} was inferred at nodes during invertebrate evolution. The maximum likelihood phylogenetic tree (see Fig. 3a) was used as a guide. The set of states at each node is ordered from most likely to least likely, excluding states with probabilities below 5%.

Supplementary Table 1. List of Primers. Related to Experimental Procedures.

Primer Name	Primer Sequence (5’-3’)
HIF1α (387-581) FWD	ATTATTGAATTCTCAGGAACTGCTTTCTAATGGTGACAACTGATCGAAGG
HIF1α (387-581) REV	ATTATTGCGGCCGCTCAGGAACTGCTTTCTAATGGTGACAACTGATCGAAGG
HIF2α P405A FWD	TGGGGTGGCAGCCAGCTGGGCCA
HIF2α P405A REV	TGGCCCAGCTGGCTGCCACCCCA
HIF2α T528M FWD	GGGATATAGGGTGCCAGCATCTCCAAGTCCAGCTCA
HIF2α T528M REV	TGAGCTGGACTTGGAGATGCTGGCACCCTATATCCC
HIF1α M561T FWD	GGGATATAGGGAGCTAACGTCTCCAAGTCTAAATCTG
HIF1α M561T REV	CAGATTTAGACTTGGAGACGTTAGCTCCCTATATCCC
VHL F91Y FWD	TCGCCGTCGTAGTTGAGCCATACGGGC
VHL F91Y REV	GCCCGTATGGCTCAACTACGACGGCGA
VHL F91W FWD	GGCTCGCCGTCCCAGTTGAGCCATACGGG
VHL F91W REV	CCCGTATGGCTCAACTGGGACGGCGAGCC
VHL F91L FWD	GCTCGCCGTCTAAGTTGAGCCATACGGGC
VHL F91L REV	GCCCGTATGGCTCAACTTAGACGGCGAGC

Supplementary Table 2. Sequence ID for HIF1α, HIF2α, and VHL. Related to Experimental Procedures.

Species Name	Common Name	HIF1 Sequence ID	Gene ID	Protein ID
Homo sapiens	Human	HIF1α Sequence ID	NM_001530.3	NP_001521.1
		HIF2α Sequence ID	U81984.1	AAB41495
		VHL Sequence ID	NM_000551.3	NP_000542.1
Macaca fascicularis	Crab-eating macaque	HIF1α Sequence ID	NM_001283896.1	NP_001270825.1
		HIF2α Sequence ID	XM_005575965.2	XP_005576022.1
		VHL Sequence ID		
Delphinapterus leucas	Beluga whale	HIF1α Sequence ID	KJ619999.1	AIB53793.1
		HIF2α Sequence ID	KX227381.1	APX43029.1
		VHL Sequence ID	XM_022564705.1	XP_022420413.1
Capra hircus	Goat	HIF1α Sequence ID	KC700026.1	AGM38929.1
		HIF2α Sequence ID	XM_018055188.1	XP_017910677.1
		VHL Sequence ID	XM_018038280.1	XP_017893769.1
Mus musculus	Mouse	HIF1α Sequence ID	AF003695.1	AAC53455.1
		HIF2α Sequence ID	BC057870.1	AAH57870.1
		VHL Sequence ID	NM_009507.4	NP_033533.1
Ictidomys tridecemlineatus	Squirrel	HIF1α Sequence ID	XM_013357468.2	XP_013212922.1
		HIF2α Sequence ID	XM_005324504.3	XP_005324561.1
		VHL Sequence ID	XM_005343104.3	XP_005343161.2
Meriones unguiculatus	Gerbil	HIF1α Sequence ID	XM_021651031.1	XP_021506706.1
		HIF2α Sequence ID	XM_021643571.1	XP_021499246.1
		VHL Sequence ID	XM_021637655.1	XP_021493330.1
Gallus gallus	Chicken	HIF1α Sequence ID	NM_204297.1	NP_989628.1
		HIF2α Sequence ID	NM_204807.2	NP_990138.1
		VHL Sequence ID	XM_414447.5	XP_414447.3
Anas platyrhynchos	Mallard	HIF1α Sequence ID	XM_005029943.3	XP_005030000.2
		HIF2α Sequence ID	XM_005009804.3	XP_005009861.2
		VHL Sequence ID		
Limosa lapponica baueri	Bar-tailed godwit	HIF1α Sequence ID	KZ506160.1	PKU41137.1
		HIF2α Sequence ID	KZ505646.1	PKU48810.1
		VHL Sequence ID		
Cyanistes caeruleus	Blue tit	HIF1α Sequence ID	XM_023927146.1	XP_023782914.1
		HIF2α Sequence ID	XM_023924504.1	XP_023780272.1
		VHL Sequence ID	XM_023935659.1	XP_023791427.1
Columba livia	Rock pigeon	HIF1α Sequence ID	AKCR02000005.1	PKK31254.1
		HIF2α Sequence ID	AKCR02000024.1	PKK26737
		VHL Sequence ID		
Phrynocephalus przewalskii	Toadhead Agama	HIF1α Sequence ID	KP696482.1	ALS35220.1
		HIF2α Sequence ID		
		VHL Sequence ID		
Phrynocephalus erythrurus	Agama	HIF1α Sequence ID	KP696483.1	ALS35221.1
		HIF2α Sequence ID	KP696480.1	ALS35218.1
		VHL Sequence ID		
Pogona vitticeps	Central bearded dragon	HIF1α Sequence ID	XM_020813439.1	XP_020669098.1
		HIF2α Sequence ID	XM_020796898.1	XP_020652557.1
		VHL Sequence ID	XM_020791430.1	XP_020647089.1
Ophiophagus hannah	King cobra	HIF1α Sequence ID	AZIM01002394.1	ETE64108.1
		HIF2α Sequence ID		
		VHL Sequence ID	AZIM01000385.1	ETE71348.1
Xenopus laevis		HIF1α Sequence ID	DQ529235.1	ABF71072.1

	African clawed frog	HIF2α Sequence ID	NM_001092249.1	NP_001085718.1
		VHL Sequence ID	KC700047.1	AHE80969.1
Xenopus tropicalis	Western clawed frog	HIF1α Sequence ID		
		HIF2α Sequence ID	NM_001005647.1	NP_001005647.1
		VHL Sequence ID		
Rana temporaria	Common frog	HIF1α Sequence ID	EU262663.1	ABY86629.1
		HIF2α Sequence ID		
		VHL Sequence ID		
Protopterus annectens	West African lungfish	HIF1α Sequence ID	JQ031040.1	AFU07559.1
		HIF2α Sequence ID		
		VHL Sequence ID		
Latimeria chalumnae	Coelacanth	HIF1α Sequence ID	XM_005986412.2	XP_005986474.1
		HIF2α Sequence ID	XM_006007491.2	XP_006007553.1
		VHL Sequence ID	XM_005987945.2	XP_005988007.1
Danio rerio	Zebrafish	HIF1α Sequence ID	NM_001310042.1	NP_001296971.1
		HIF2α Sequence ID	NM_001039806.2	NP_001034895.2
		VHL Sequence ID	NM_001080684.1	NP_001074153.1
Oncorhynchus tshawytscha	Chinook Salmon	HIF1α Sequence ID	XM_024437278.1	XP_024293046.1
		HIF2α Sequence ID	XM_024388216.1	XP_024243984.1
		VHL Sequence ID	XM_024407786.1	XP_024263554.1
Cyprinus carpio	Common carp	HIF1α Sequence ID	EU144225.1	ABV59209.1
		HIF2α Sequence ID	XM_019113102.1	XP_018968647.1
		VHL Sequence ID	XM_019077133.1	XP_018932678.1
Acipenser gueldenstaedtii	Russian sturgeon	HIF1α Sequence ID	EF100701.1	ABO26712.1
		HIF2α Sequence ID		
		VHL Sequence ID		
Mustelus canis	Smooth dogfish	HIF1α Sequence ID	EU262662.1	ABY86628.1
		HIF2α Sequence ID		
		VHL Sequence ID		
Hemiscyllium ocellatum	Epaulette shark	HIF1α Sequence ID	EU262661.1	ABY86627.1
		HIF2α Sequence ID	GQ152300.1	ADD59897.1
		VHL Sequence ID		
Rhincodon typus	Whale shark	HIF1α Sequence ID	XM_020515091.1	XP_020370680.1
		HIF2α Sequence ID	XM_020510059.1	XP_020365648.1
		VHL Sequence ID	XM_020532548.1	XP_020388137.1
Callorhinchus milii	Elephant shark	HIF1α Sequence ID	XM_007903838.1	XP_007902029.1
		HIF2α Sequence ID	XM_007896863.1	XP_007895054.1
		VHL Sequence ID	XM_007904937.1	XP_007903128.1
Petromyzon marinus	Lamprey	HIF1α Sequence ID	ENSPMAG00000000126	ENSPMAT00000000148.1
		HIF2α Sequence ID	ENSPMAG00000009272	ENSPMAT00000010244.1
		VHL Sequence ID	ENSPMAG00000009828.1	ENSPMAP00000010802.1
Saccoglossus kowalevskii	Acorn worm	HIF1α Sequence ID	XM_002733741.3	XP_002733787.2
		HIF2α Sequence ID		
		VHL Sequence ID	XM_002733273.2	XP_002733319.1
Strongylocentrotus purpuratus	Pacific purple sea urchin	HIF1α Sequence ID	KX786251.1	ASL69982.1
		VHL Sequence ID	KX786255.1	ASL69986.1
Acanthaster planci	Crown-of-thorns starfish	HIF1α Sequence ID	XM_022242264.1	XP_022097956.1
		VHL Sequence ID	XM_022236676.1	XP_022092368.1
Haliotis diversicolor	Sea snail	HIF1α Sequence ID	KC149963.1	AGE97172.1
		VHL Sequence ID		
Crassostrea gigas	Pacific oyster	HIF1α Sequence ID	NM_001305337.1	NP_001292266.1
		VHL Sequence ID	XM_011431794.2	XP_011430096.1

Crassostrea virginica	Eastern oyster	HIF1α Sequence ID	HM441076.1	AED87588.1
		VHL Sequence ID	XM_022484237.1	XP_022339945.1
Mizuhopecten yessoensis	Yesso scallop	HIF1α Sequence ID	XM_021497943.1	XP_021353618.1
		VHL Sequence ID	XM_021485147.1	XP_021340822.1
Lingula anatina	Lamp shell	HIF1α Sequence ID	XM_013531134.2	XP_013386588.1
		VHL Sequence ID	XM_013557922.1	XP_013413376.1
Orussus abietinus	Parasitic wood wasp	HIF1α Sequence ID	XM_012420115.2	XP_012275538.1
		VHL Sequence ID	XM_012420856.1	XP_012276279.1
Nasonia vitripennis	Jewel wasp	HIF1α Sequence ID		
		VHL Sequence ID	XM_016983273.1	XP_016838762.1
Bombus terrestris	Buff-tailed bumblebee	HIF1α Sequence ID		
		VHL Sequence ID	XM_003399514.3	XP_003399562.1
Trachymyrmex cornetzi	Ant	HIF1α Sequence ID	KQ978957.1	KYN27213.1
		VHL Sequence ID	XM_018510151.1	XP_018365653.1
Nilaparvata lugens	Brown planthopper	HIF1α Sequence ID	XM_022331880.1	XP_022187572.1
		VHL Sequence ID		
Onthophagus taurus	Dung beetle	HIF1α Sequence ID	XM_023055980.1	XP_022911748.1
		VHL Sequence ID		
Ixodes scapularis	Black-legged tick	HIF1α Sequence ID		
		VHL Sequence ID	XM_002407200.1	XP_002407244.1
Limulus polyphemus	Atlantic horseshoe crab	HIF1α Sequence ID	XM_013921719.2	XP_013777173.1
		VHL Sequence ID		
Daphnia magna	Waterflea	HIF1α Sequence ID	AB425958.1	BAG69568.1
		VHL Sequence ID	LRGB01000915.1	KZS15206.1
Daphnia pulex	Waterflea	HIF1α Sequence ID	GL732533.1	EFX84860.1
		VHL Sequence ID	GL732523.1	EFX89933.1
Caenorhabditis elegans	Roundworm	HIF1α Sequence ID	NM_075607.5	NP_508008.4
		VHL Sequence ID	NM_077488.3	NP_509889.1
Ascaris suum	Pig roundworm	HIF1α Sequence ID	AB520828.1	BAJ17131.1
		VHL Sequence ID		
Pristionchus pacificus	Roundworm	HIF1α Sequence ID	ABKE03000053.1	PDM75007.1
		VHL Sequence ID		
Exaiptasia pallida	sea anemone	HIF1α Sequence ID	LJWW01000106.1	KXJ20783.1
		VHL Sequence ID	XM_021061147.1	XP_020916806.1
Nematostella vectensis	Starlet sea anemone	HIF1α Sequence ID	KJ411881.1	AII22158.1
		VHL Sequence ID		
Orbicella faveolata	mountainous star coral	HIF1α Sequence ID	XM_020774709.1	XP_020630368.1
		VHL Sequence ID	XM_020746540.1	XP_020602199.1
Stylophora pistillata	Smooth cauliflower coral	HIF1α Sequence ID	XM_022941810.1	XP_022797545.1
		VHL Sequence ID	XM_022948633.1	XP_022804368.1
Acropora digitifera	Coral	HIF1α Sequence ID		
		VHL Sequence ID	XM_015898670.1	XP_015754156.1
Trichoplax adhaerens	Tablet animal	HIF1α Sequence ID	JQ844128.1	AFM37575.1
		VHL Sequence ID		Triad1P7508
