# Supplementary material for: Evolution of metazoan oxygen-sensing involved a conserved divergence of VHL affinity for HIF1α and HIF2α
Source: Nat Commun. 2019 Jul 23;10:3293. doi: 10.1038/s41467-019-11149-1 (PMC6650433; doi:10.1038/s41467-019-11149-1)
Supplement: Supplementary file 3 — Reporting Summary [file 41467_2019_11149_MOESM3_ESM.pdf]

nature research

Corresponding author(s): *Double-blind peer review submissions: write DBPR and your manuscript number here instead of author names.*

Last updated by author(s): *YYYY-MM-DD*

Reporting Summary

Nature Research wishes to improve the reproducibility of the work that we publish. This form provides structure for consistency and transparency in reporting. For further information on Nature Research policies, see Authors & Referees and the Editorial Policy Checklist.

Statistics

For all statistical analyses, confirm that the following items are present in the figure legend, table legend, main text, or Methods section.

n/a	Confirmed	
☐	☒	The exact sample size (*n*) for each experimental group/condition, given as a discrete number and unit of measurement
☐	☒	A statement on whether measurements were taken from distinct samples or whether the same sample was measured repeatedly
☐	☒	The statistical test(s) used AND whether they are one- or two-sided<br>*Only common tests should be described solely by name; describe more complex techniques in the Methods section.*
☐	☒	A description of all covariates tested
☐	☒	A description of any assumptions or corrections, such as tests of normality and adjustment for multiple comparisons
☐	☒	A full description of the statistical parameters including central tendency (e.g. means) or other basic estimates (e.g. regression coefficient) AND variation (e.g. standard deviation) or associated estimates of uncertainty (e.g. confidence intervals)
☒	☐	For null hypothesis testing, the test statistic (e.g. *F*, *t*, *r*) with confidence intervals, effect sizes, degrees of freedom and *P* value noted<br>*Give P values as exact values whenever suitable.*
☒	☐	For Bayesian analysis, information on the choice of priors and Markov chain Monte Carlo settings
☒	☐	For hierarchical and complex designs, identification of the appropriate level for tests and full reporting of outcomes
☒	☐	Estimates of effect sizes (e.g. Cohen's *d*, Pearson's *r*), indicating how they were calculated

Our web collection on statistics for biologists contains articles on many of the points above.

Software and code

Policy information about availability of computer code

Data collection	N/A
Data analysis	Mega7 software was used to identify annotated HIF2α, HIF1α, and VHL sequences. The GUIDANCE2 webserver was used for sequence alignment. ESPript3 was used to visualize sequence alignments. PhyML 3.0 was used to generate a phylogenetic tree. Figtree was used to visualize the phylogenetic tree. The HyPhy package, accessed via Mega7 software, was used to estimate synonymous and non-synonymous mutation rates. Consurf was used to generate visual representations of HIF1α and VHL evolutionary conservation at the amino acid level. Mega7 software was used to reconstruct ancestral amino acid sequences. References for these programs are included in the materials and methods section.

For manuscripts utilizing custom algorithms or software that are central to the research but not yet described in published literature, software must be made available to editors/reviewers. We strongly encourage code deposition in a community repository (e.g. GitHub). See the Nature Research guidelines for submitting code & software for further information.

Data

Policy information about availability of data

All manuscripts must include a data availability statement. This statement should provide the following information, where applicable:

- Accession codes, unique identifiers, or web links for publicly available datasets
- A list of figures that have associated raw data
- A description of any restrictions on data availability

Source data for Figures 1a, 1b, 2b, 2c, 2d, 2e, 3a, 3b, 3c, 4b, 4c, 4d, 4e, 5b, 5c, 5d, 5e, and supplementary figures 1a, 1b, 1c, 2a, 2b, 2c, 4a, 4b, 5, 6b are provided as a Source Data file. All data are available from the corresponding author upon reasonable request.

Field-specific reporting

Please select the one below that is the best fit for your research. If you are not sure, read the appropriate sections before making your selection.

☒ Life sciences ☐ Behavioural & social sciences ☐ Ecological, evolutionary & environmental sciences

For a reference copy of the document with all sections, see nature.com/documents/nr-reporting-summary-flat.pdf

Life sciences study design

All studies must disclose on these points even when the disclosure is negative.

Sample size	Experiments were replicated three times in keeping with scientific convention.
Data exclusions	No data was excluded from analysis.
Replication	We have replicated our interaction experiments, as reported in the manuscript, with biolayer interferometry (BLI), surface plasmon reference (SPR), pull-down, and co-immunoprecipitation studies. All experiments show the same trend. The reported kinetic values differ by less than one order of magnitude between SPR and BLI, which we attribute to different temperature. See methods for more information.
Randomization	N/A
Blinding	Investigators were not blinded. Not applicable to biochemical and biophysical experiments, with quantitative and objective readouts.

Reporting for specific materials, systems and methods

We require information from authors about some types of materials, experimental systems and methods used in many studies. Here, indicate whether each material, system or method listed is relevant to your study. If you are not sure if a list item applies to your research, read the appropriate section before selecting a response.

Materials & experimental systems

n/a	Involved in the study	
☐	☒	Antibodies
☒	☐	Eukaryotic cell lines
☒	☐	Palaeontology
☒	☐	Animals and other organisms
☒	☐	Human research participants
☒	☐	Clinical data

Methods

n/a	Involved in the study	
☒	☐	ChIP-seq
☒	☐	Flow cytometry
☒	☐	MRI-based neuroimaging

Antibodies

Antibodies used	A catalog number and working dilution is provided for every antibody. See methods section under heading "Antibodies"
Validation	See Figure 1a. The specificity of the HA antibody was tested by including an empty vector (HA alone) control.
